# Artificial Intelligence in Dermatopathology: An Update and Review of the Current Literature

**DOI:** 10.3390/diagnostics16111702

**Published:** 2026-06-01

**Authors:** Ala’ Abu-Dayeh, Gerardo Cazzato, Alessio Giubellino

**Affiliations:** 1 Department of Laboratory Medicine and Pathology, University of Minnesota, Minneapolis, MN 55414, USA; abuda008@umn.edu; 2 Section of Molecular Pathology, Department of Precision and Regenerative Medicine and Ionian Area (DiMePRe-J), University of Bari Aldo Moro, 70121 Bari, Italy; gerardo.cazzato@uniba.it; 3 Masonic Cancer Center, University of Minnesota, Minneapolis, MN 55414, USA

**Keywords:** artificial intelligence, dermatopathology, machine learning, deep learning, digital pathology

## Abstract

Artificial intelligence (AI) has pervaded many fields of medicine in the last few years on the wave of similar changes in other disciplines. Adoption of AI-driven technologies will progress in pathology in the years to come and will also transform our subspecialty of dermatopathology. From the adoption of AI in teaching to its use in clinical practice and in advancing our field through improved research capabilities, we expect a great deal of changes that will hopefully improve our assessment of tissue sections for several cutaneous pathologies. In this review, we offer an overview of where the use of these tools currently stands in dermatopathology and the potential directions that will transform the way we practice and do research. We cover AI’s role in diagnosing various skin conditions, such as melanocytic lesions and other cutaneous skin cancers, and inflammatory dermatoses. The review further covers AI’s contributions to workflow automation, like mitotic figure detection and counting, predictive analytics (e.g., melanoma prognosis), and educational tools (e.g., AI-driven simulators). It also addresses critical technical aspects, including data curation, algorithm development, and model validation. We aim to provide a comprehensive overview of how AI is transforming dermatopathology, from diagnosis and prognosis to education and clinical integration.

## 1. Introduction

### 1.1. Overview of Dermatopathology and Its Challenges

Dermatopathology, as a joint subspecialty of pathology and dermatology, focuses on the microscopic examination of skin diseases. Among the challenges in this field are the vast range of skin pathologies, with more than 2000 recognized skin diseases spanning from inflammatory dermatoses, infections, deposit disease, and neoplastic lesions [1]. Accurate diagnosis often requires close clinical correlation to effectively interpret the microscopic findings for this large variety of ailments.

In addition, the presence of overlapping histopathological features in many skin diseases, particularly among inflammatory dermatoses and melanocytic lesions, accounts for inter-observer discordance and variability in dermatopathological diagnoses, which, in turn, affects diagnostic reliability and clinical outcomes [2].

Moreover, there is a recent surge in the volume of work in dermatopathology due to a rising number of skin diseases and greater public awareness of skin cancer [3]. The rising volume of skin specimens, together with the complexity of some of these cases, places additional pressure on dermatopathologists to make accurate and timely diagnoses. Some of the challenges include the need for timely clinical decisions, particularly in cases where diagnostic delays directly affect patient outcomes. Second, there is a shortage of dermatopathologists in many regions, resulting in pathologist fatigue and burnout [4].

### 1.2. Introduction to Artificial Intelligence (AI) and Machine Learning (ML)

#### 1.2.1. Definitions and Fundamental Concepts

Artificial intelligence (AI) involves developing computer systems that can perform tasks typically requiring human intelligence, such as understanding information, reasoning, and decision-making [5]. A subfield of AI is machine learning (ML), which focuses on creating algorithms and models enabling machines to learn from experience and data and improve task performance over time without being specifically programmed for every task [6]. In simple terms, instead of teaching a computer to do something in particular, you give it data and let it learn to recognize patterns or make predictions from the data. ML is subclassified into three groups: supervised learning, unsupervised learning, and reinforcement learning [7]. In supervised learning, the algorithm is trained on labeled data, and it learns to make predictions based on that. In unsupervised learning, the algorithm works with unlabeled data to find hidden patterns. In reinforcement learning, the algorithm learns by trial and error [7]. Deep learning is a subset of ML that uses neural networks with multiple layers to achieve high levels of accuracy in image, sound, and language identification. These networks mimic how neurons are connected in the human brain, organizing information in layered arrangements [6]. Convolutional neural networks (CNNs) are a type of artificial neural network that are designed to detect and process images and visual data. When an image is input into a CNN, the image appears as a pixel grid. The CNN then applies filters to detect simple patterns. As the network goes deeper through multiple layers, it gradually combines these simple patterns to recognize more complex shapes and features. At the final stage, the network utilizes this information to label or interpret the image, such as identifying it with a precise diagnosis, e.g., as a melanoma [8].

#### 1.2.2. Historical Context of AI in Medicine

The history of AI in medicine dates back to the 1950s [9], when early systems attempted to support clinical decision-making. However, these early efforts were limited by inability to process complex clinical data in real time [10]. As technology advanced, especially with the development of deep learning, AI systems became more sophisticated and capable of analyzing large datasets, identifying patterns, and improving diagnostic precision. This shift enabled more clinical applications across different specialties, including radiology, pathology, and gastroenterology [10]. In dermatopathology, the first use of AI dates back to 1987, when the TEGUMENT system was developed. It used a decision tree to help dermatologists in forming a differential diagnosis after reviewing histologic slides under light microscopy. It achieved an accuracy of 91.9%; however, it relied on restructuring traditional medical data to fit into the system, making it a tool to help in diagnosis rather than an independent machine-driven process [11].

### 1.3. Rationale for AI Integration in Dermatopathology

The integration of AI into clinical dermatopathology offers several potential advantages but is also associated with important challenges and limitations (Table 1).

#### 1.3.1. Potential to Enhance Diagnostic Accuracy and Efficiency

CNNs have shown good capabilities in recognizing subtle patterns in histopathology slides that can be missed by human eyes. AI can help in reducing diagnostic errors and improving consistency, for example, by its ability to help in the classification of different lesions and detecting mitoses. AI assistance could also save time, especially in high-volume settings, as it can process large volumes of slides in a short period [12].

#### 1.3.2. Role in Addressing Workforce Challenges

AI can help alleviate the burden caused by the shortage of dermatopathologists by, for example, triaging cases, functioning as a second reader, producing consistent reports, and standardizing interpretations across institutions. Overall, integrating AI into dermatopathology holds great promise for improving diagnostic quality [13].

## 2. Review Methodology

This manuscript is a narrative review of the literature. A non-systematic search of the PubMed database was performed to identify relevant studies published between 1985 and 2026 using combinations of keywords related to “dermatopathology”, “histopathology”, “artificial intelligence,” “machine learning”, “deep learning”, and “multimodal large language model”. Additional articles were identified through manual screening of references from selected publications. Studies were included based on their relevance to AI applications in dermatopathology, with emphasis on histopathology-based investigations. Given the narrative nature of this review, no formal inclusion/exclusion criteria or systematic quality appraisal framework was applied.

## 3. AI Applications in Dermatopathology: Current Landscape

**Figure 1:**
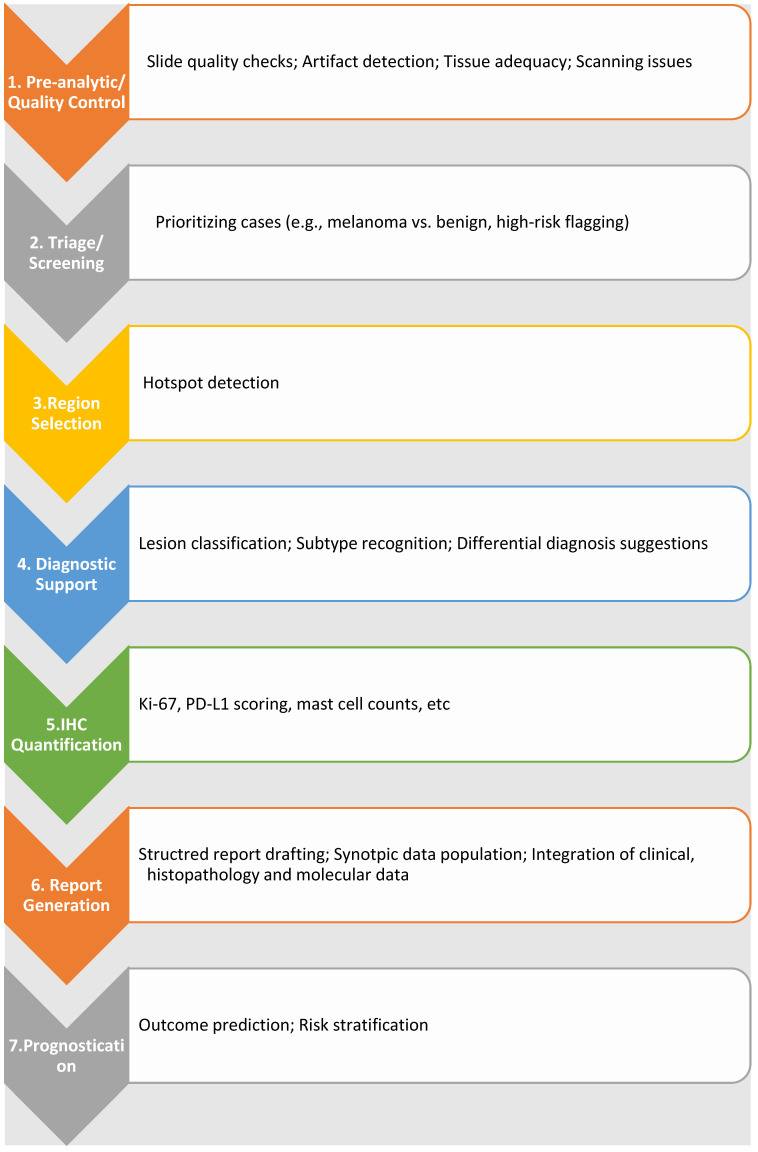
Artificial intelligence applications mapped to the clinical workflow in dermatopathology. Artificial intelligence may support multiple stages of the dermatopathology sign-out process, beginning with pre-analytic quality control and case triage, followed by hotspot identification, diagnostic decision support, quantitative immunohistochemical, integrated reporting, and prognostication.

Key applications of AI in dermatopathology are summarized in Figure 1, which maps AI tools across the clinical workflow, and Table 2, which provides a detailed overview of specific AI applications in dermatopathology.

### 3.1. Diagnostic Support for Specific Skin Conditions

#### 3.1.1. Melanocytic Lesions (Nevi vs. Melanoma)

*a.*  *Discrimination between benign nevi and malignant melanoma*

Several studies have investigated CNNs for nevi vs. melanoma discrimination. Hekler et al. explored the use of ResNet50 CNNs to distinguish between benign nevi and malignant melanoma on histopathological slides [14]. The researchers trained a model on 595 hematoxylin and eosin-stained images of melanocytic lesions (350 nevi and 345 melanomas). The model’s performance was directly compared with that of 11 practicing pathologists using a separate test set of 100 image sections (1:1 ratio of melanomas to nevi). The overall disagreement with the pathologist was 18% for melanoma cases and 20% for nevi. While the results suggest that deep learning models can match or exceed human-level performance under certain conditions, the study also had limitations, including the algorithm’s binary classification approach; unlike pathologists, who must consider a wide range of differential diagnoses, the model can only assess whether a lesion is more likely to be a nevus or melanoma. This is an important limitation, especially in melanocytic pathology, where many lesions fall within a spectrum that includes intermediate lesions. Another study to discriminate between benign nevi and malignant melanoma was conducted by Brinker et al. [15]. Researchers compared the performance of CNN with 18 internationally recognized expert dermatopathologists in classifying melanomas versus nevi using hematoxylin and eosin WSIs. The CNNs were trained on WSIs of 50 melanomas and 50 nevi. The ensemble CNN matched or slightly exceeded expert diagnostic performance, achieving AUCs as high as 0.97. Its performance closely aligned with the average sensitivity and specificity of international experts. These results highlight the potential for well-trained CNNs to assist or support expert-level dermatopathological diagnosis.

*b.*  *Recognition of specific melanoma subtypes*

Nevoid melanoma (NM) is a rare melanoma subtype and one of the most diagnostically challenging melanoma subtypes. Cazzato and colleagues proposed the application of a fast random forest (FRF) algorithm to support the early screening of NM [16]. They developed a business process modeling and notation (BPMN) workflow, incorporating the FRF algorithm into the diagnostic pipeline. Using image-based cluster analysis, the algorithm flagged potential regions within biopsies, suggesting that FRF-based screening could enhance diagnostic accuracy in challenging NM cases.

*c.*  *Performance metrics (accuracy, sensitivity, specificity) compared to human experts*

Deep learning algorithms have shown strong performance in the histopathologic evaluation of melanocytic lesions, often comparable to or exceeding human experts: for example, Brinker et al., 2022 [15], used an ensemble CNN with annotation and achieved 94% mean sensitivity, 90% specificity, and 92% accuracy in classifying melanoma versus benign nevi, while dermatopathologists achieved a mean sensitivity of 88.88%, specificity of 91.77%, and accuracy of 90.33%.

In another setting, Hekler et al., 2019 [17], using a CNN model, achieved a mean sensitivity of 76%, specificity of 60%, and accuracy of 68% in classification of melanoma histologic images. In comparison, the pathologists achieved a mean sensitivity of 51.8%, specificity of 66.5%, and accuracy of 59.2%.

*d.*  *Examples of successful deep learning models (e.g., CNNs like ResNet50)*

Several deep learning models have been applied successfully to melanocytic lesions. One of the most commonly used is ResNet50, a CNN model well known for its success in image classification and used in classification of many cutaneous lesions, including melanoma classification, achieving a high diagnostic accuracy [14,18]. Among other models frequently used are U-Net variants, usually employed for image segmentation tasks such as identifying tumor regions within tissue slides before classifications [19,20]. Hybrid models are utilized in certain studies to couple CNN-based feature extraction with traditional machine learning classifiers, thereby improving diagnostic performance [21].

#### 3.1.2. Non-Melanoma Skin Cancers (e.g., Basal Cell Carcinoma, Squamous Cell Carcinoma)

*a.*  *Detection and classification on whole-slide images (WSIs)*

One of the earlier applications of deep learning applied to basal cell carcinoma (BCC) was introduced by Roa et al. The authors developed a deep learning framework designed for automatic image representation and classification, capable of analyzing histopathological images without requiring extensive handcrafted feature engineering. Evaluated on approximately 1400 image patches drawn from annotated tissue regions, the model achieved a balanced accuracy of 91.4%, outperforming conventional feature-based approaches [22]. Duschner et al. applied a U-Net-based model for automated detection and subtyping of BCC in a routine diagnostic setting. Across three dermatopathology centers, the AI model achieved sensitivities ranging from ~97.7% to 98.6% and specificities around 96.8–98.7%. Beyond simple detection, the system also provided tumor subtyping and thickness estimates, underscoring the potential of AI to improve diagnostic efficiency [19]. Using a multi-stage deep learning pipeline to segment tumor and nuclei, Lan et al. extracted interpretable morphologic and microenvironment features to distinguish BCC from trichoepithelioma (TE) and stratify BCC into high- vs. low-risk subtypes, achieving high AUCs across internal and external cohorts [23].

*b.*  *Identification of tumor margins*

Since the successful management of solid tumors requires complete surgical excision, and conventional radial margin assessment may fail to detect residual disease, a 2024 study introduced an artificial intelligence platform, ArcticAI, which is designed to enhance intraoperative margin assessment during Mohs micrographic surgery for BCC [24]. This system combines deep learning and histologic tumor mapping to streamline the surgical workflow. It automates tissue grossing recommendations, accurately identifies tumor presence on whole slide images, and maps these findings back to the surgical site. This integrated approach improves the speed and completeness of intraoperative margin evaluations for BCC excision.

Another study applied machine learning to audit surgical margins after curative excision of facial non-melanoma skin cancers [25]. Analyzing 3354 cases, the authors found positive margins in 15–21% of the cases. Machine learning models incorporating patient and tumor characteristics achieved good predictive performance with moderate accuracy, offering a method for risk-adjusted evaluation of surgical performance and enabling fair comparisons across treatment centers.

*c.*  *Examples of AI-driven tools in Mohs micrographic surgery*

Mohs microscopic surgery is a specialized technique for treating skin cancer in which thin layers of skin with cancer are removed and examined under the microscope until only cancer-free tissue remains [26]. A retrospective study developed a deep learning algorithm to detect cutaneous squamous cell carcinoma (cSCC) on frozen sections during Mohs micrographic surgery. Researchers scanned and annotated slides to distinguish between tumor, benign structures, and inflammation and then trained a CNN to extract histomorphological features associated with cSCC [27]. The model showed good accuracy for moderate to poorly differentiated cSCC but struggled with well-differentiated cases, highlighting the need to integrate tissue architecture for improved performance in real-time margin assessment.

#### 3.1.3. Inflammatory Dermatoses

*a.*  *Challenges in classification due to overlapping features*

As mentioned above, AI has shown strong progress in dermatopathology, particularly for neoplastic diseases such as BCC. However, its role in inflammatory skin diseases remains limited [28]. This limitation arises because inflammatory conditions often exhibit overlapping features and heterogenous morphology across disease stages. Furthermore, accurate diagnosis often requires clinical context that current AI systems cannot integrate.

*b.*  *Emerging applications and limitations*

A study by Pal et al. developed a U-shaped deep CNN to segment psoriasis skin biopsy images, effectively distinguishing the epidermis and dermis despite challenges like uneven staining and complex cellularity [29]. The model outperformed traditional feature-based methods, demonstrating the potential of deep learning for analyzing inflammatory skin diseases.

In a different setting, Bao et al. introduced an AI-based approach to classify subtypes of superficial perivascular dermatitis—specifically psoriasiform, spongiotic, and interface types—by analyzing histopathological images [30]. The researchers utilized a dataset of 3954 images from 327 cases, employing a cascaded deep learning framework that combined pixel-level segmentation with subtype classification. The model achieved an overall accuracy of 85.24%, significantly outperforming a baseline model without recognition capabilities, which had an accuracy of 71.35%. The study demonstrated that AI can effectively assist in identifying key pathological features and classifying inflammatory skin conditions, potentially aiding dermatopathologists in diagnosing and differentiating between subtypes of superficial perivascular dermatitis.

#### 3.1.4. Adnexal Tumors and Other Rare Entities

*a.*  *Current research and the need for larger datasets*

AI has shown also considerable promise in the histological classification of adnexal tumors, a group of skin neoplasms with overlapping features where other ancillary studies, like immunohistochemistry, are of limited utility. A recent study developed a CNN to classify 14 different cutaneous adnexal tumors using histopathological images [31]. The model achieved an overall classification accuracy of 89.92% across 248 samples. Notably, it successfully distinguished some rare tumors within this category from more common skin lesions like BCC and seborrheic keratosis, even with limited training data (fewer than 50 cases per class). The study underscores the potential of AI to assist dermatopathologists in diagnosing less common skin tumors, enhancing diagnostic efficiency and accuracy. However, the application of AI in this domain is hindered by the scarcity of large, annotated datasets.

### 3.2. Workflow Optimization and Automation

#### 3.2.1. Automated Detection of Mitotic Figures

Sturm et al. evaluated an AI-based mitosis detection tool, which was originally designed for breast cancer, on 99 digitized melanocytic lesion slides (including 10 nevoid melanomas) [32]. Eight pathologists reviewed cases twice: first without algorithm annotations and then with algorithm-assisted mitosis highlighting. While overall diagnostic concordance with expert consensus was similar (~90%), in nevoid melanoma cases, the algorithm improved concordance from 68% to 75%. The AI flagged numerous false positives (e.g., pigmented or keratinocyte nuclei), underscoring the need for cautious interpretation. These findings suggest that mitosis detection algorithms may be helpful in particularly challenging cases like nevoid melanoma but offer limited benefit in routine lesion interpretation. Further development of similar models may hopefully lead to a more reliable platform for potential deployment in routine practice.

#### 3.2.2. Identification of Hotspots for Further Review

AI-based identification of hotspots enables automated detection of regions within tissue sections that are most likely to contain diagnostically significant features such as clusters of atypical melanocytes or high mitotic activity. Hotspot detection helps prioritize slide review, ensuring pathologists focus on the most informative regions first [12]. Current methods rely on attention-based multiple instance learning (MIL) and transformers, saliency-inference techniques for small lesions, and uncertainty-aware heatmaps that flag areas requiring human verification [33,34]. These approaches can reduce review time and improve safety but face challenges such as spurious attention to artifacts, sensitivity-specificity trade-offs in small-lesion detection, and overconfidence on out-of-distribution detection [33,35,36]. Clinically meaningful performance requires that hotspot detection reliably highlights diagnostically relevant areas (e.g., small foci of invasion or mitotic activity) while avoiding distraction by artifacts such as tissue folds or staining irregularities. Key failure modes include missing focal invasive melanoma or overcalling non-neoplastic artifacts as hotspots. Validation strategies vary and include comparison with pathologist-annotated regions, lesion-level ground truth, and external dataset testing. Methods that demonstrate consistent concordance with expert-annotated diagnostic regions and maintain performance across external cohorts are closer to clinical deployment, whereas systems validated only on slide-level labels remain more vulnerable to clinically significant localization errors.

#### 3.2.3. Quantification of Immunohistochemical Stains

AI-based quantification of immunohistochemical (IHC) stains in dermatopathology offers a reproducible alternative to manual scoring, which is often limited by interobserver variability. Deep learning algorithms applied to WSI can identify positive versus negative cells and measure staining intensity. In dermatopathology, applications include assessing proliferation markers (e.g., Ki-67) in melanocytic lesions [37], quantifying lineage markers (Melan-A, SOX10) [38] for melanocytic lesion evaluation, and analyzing immune-related markers (CD3, CD8, PD-L1) [39,40] in both cutaneous tumors and inflammatory dermatoses. A study by Emmanuel et al. evaluated two methods for quantifying CD8+ T cells in skin biopsies from patients with psoriasis: manual counting using Adobe Photoshop and automated analysis using QuPath [39]. The study found that normalizing cell counts to epidermal length provided more consistent results than normalization to area, particularly when comparing lesional and non-lesional skin. Bland–Altman analysis demonstrated good agreement between the two methods, suggesting that both are viable for quantifying inflammatory cell densities in skin biopsies. A multicenter study evaluated a ResNet classifier trained on Melan A IHC to detect melanomas, achieving area under the receiver operating characteristic curves (AUROCs) of 0.82 and 0.74 on external datasets; combining Melan A and H&E images further improved performance (AUROC of 0.85 and 0.81) [38]. For ensuring reproducibility of results and comparability between institutions, future research should define and document important technical parameters used in AI-based IHC quantification workflows. Such parameters include the type of the antibody stain used, whole-slide scanner type and image resolution, color normalization and thresholding methods, cell segmentation algorithms, normalization strategy (e.g., epidermal length versus tissue area), and interobserver or intraplatform agreement measurements. Establishing minimal reporting standards for AI-assisted IHC analysis may improve cross-center validation and support broader clinical implementation.

#### 3.2.4. Digital Slide Management and Annotation

Digital slide management and annotation are important for the adoption of WSIs, supporting clinical diagnosis, education, and AI development [41]. Management platforms enable efficient storage, retrieval, and integration of WSIs with pathology reports and facilitate teledermatopathology [42]. Annotation provides critical ground truth for supervised learning but is limited by time demands and inter-observer variability [43]. Annotation variability represents a major limitation to model reliability and generalizability. In dermatopathology, particularly in melanocytic lesions, even expert dermatopathologists may disagree on diagnostic classification because of overlapping morphologic features and subjective interpretation. As a result, inconsistent annotations may introduce significant variability into training datasets, causing models to learn conflicting patterns and reducing diagnostic performance, reproducibility, and external validity. Emerging solutions include standardized open-source tools (e.g., QuPath) [44], collaborative multi-institutional annotation efforts, and AI-assisted annotation workflows that reduce manual burden [45]. Weak labels, such as slide-level diagnoses derived from pathology reports, may be good for broad slide classification tasks or pre-screening. In contrast, region-of-interest (ROI)-level annotations remain essential for localization and segmentation tasks, including tumor boundary identification and mitotic figure detection [46,47].

### 3.3. Predictive Analytics and Prognostication

#### 3.3.1. Predicting Disease-Specific Survival in Melanoma

AI-driven predictive analytics are increasingly applied to prognostication in melanoma, where models are trained on histopathology can predict outcomes such as disease-specific survival by quantifying features like tumor thickness, mitotic rate, lymphocytic infiltration, and nuclear morphology [48]. Recent studies have shown that deep learning applied to digitized melanoma slides can stratify patients into risk categories comparable to or exceeding traditional histopathologic criteria, supporting its role in outcome prediction [49]. However, many of the available models remain investigational and have not yet achieved widespread clinical validation. Before integration of these models into routine clinical practice, further validation using large multi-center external cohorts, prospective studies, calibration analyses, and assessments of real-world clinical utility will be necessary to establish reproducibility, generalizability, and meaningful impact on patient management.

#### 3.3.2. Integration of Histological, Genetic, and Clinical Data (Multimodal AI)

Beyond histology alone, multimodal AI frameworks that integrate WSIs with genomic, transcriptomic, and clinical data have demonstrated improved prognostic accuracy, capturing both morphologic and molecular heterogeneity [50]. These approaches aim to provide more personalized risk assessments, inform therapeutic decision-making, and identify high-risk subgroups for closer follow-up and or adjuvant therapy.

### 3.4. Educational Tools and Training

#### 3.4.1. AI-Driven Simulators for Skill Refinement

AI is being integrated into interactive simulators and case-based learning platforms. Interactive platforms now exist where trainees can practice diagnosing cases in virtual environments and receive instant feedback [51,52]. One such resource, Hypertext Atlas of Dermatopathology, offers over 3000 annotated, high-resolution images with clinical and microscopic descriptions, enabling learners to narrow or broaden differential diagnoses through linked features and chapters [53]. Building on this concept, SlideTutor was developed as an intelligent tutoring system incorporating a virtual microscope and WSIs. It provides step-by-step feedback to guide learners of feature recognition, differential diagnosis, and final decision-making, closely mimicking one-on-one instruction [54]. ReportTutor is an intelligent tutoring system that was developed to combine a virtual microscope, WSIs, and a natural language interface to assist learners in improving the quality of their diagnostic reports and provide tailored feedback on accuracy and overall structure [55].

#### 3.4.2. Exposure to Diverse and Challenging Cases

AI systems can present diagnostically diverse material—including rare or high-complexity cases—that might be rarely encountered in routine teaching, thus broadening the learner’s exposure spectrum [8]. CAP “AI Playground” is a tool powered by PathPresenter that allows pathologists and trainees to explore a variety of real-world AI models applied to WSI, promoting familiarization with challenging case.

Despite these promising applications, educationally based AI systems should be evaluated using objective educational and clinical outcome measures. Important metrics include improvements in diagnostic accuracy, report quality, retention of information over time, avoidance of diagnostic error patterns, and calibration of diagnostic confidence based on pre- and post-training evaluation. Moreover, different types of learners, including medical students, pathology residents, fellows, and practicing pathologists, will experience differences in outcomes. Careful assessment is also needed to determine whether prolonged AI-assisted learning could lead to increased dependence on algorithmic suggestions or reduced independent diagnostic reasoning, highlighting the importance of maintaining balanced human oversight during training.

**Table 2:** Applications of artificial intelligence (AI) in dermatopathology.

Application Area	AI Tool	Clinical Use	Dataset Example	Key Findings	References
**A.** **Diagnosis**	Convolutional neural network (CNN): ResNet50, and ensemble CNNs	Discrimination between benign nevi and malignant melanoma	Hematoxylin and eosin (H&E)-stained slides, 595 images (350 nevi, 345 melanomas)	ResNet50 CNNs can match/exceed pathologists; binary classification limits consideration of intermediate lesions	[14]
			Whole-slide image (WSI) of 50 melanomas and 50 nevi	Ensemble CNN matched or slightly exceeded diagnostic performance	[15]
	Fast random forest (FRF) algorithm	Early screening of nevoid melanoma	18 histopathological photomicrographs of nevoid melanoma	Enhances screening for rare melanoma subtypes	[16]
	Deep learning framework; U-Net	Detection and subtyping of basal cell carcinoma (BCC)	1400 image patches; multi-center BCC cohorts	Automates detection and subtyping; reduces need for handcrafted features	[22]
	Deep learning platform (ArcticAI)	Tumor margin identification during Mohs surgery	194 BCC patients, with 178 cases used for histological analysis generating 351 WSIs	Improves intraoperative margin assessment; integrates with surgical workflow	[24]
	U-shaped deep CNN; cascaded CNN framework	Classification of inflammatory dermatoses	90 annotated psoriasis skin biopsy images	Effective for segmenting epidermis/dermis; overlapping features and lack of clinical context remain challenges	[29]
			Psoriasis: skin biopsy images; superficial perivascular dermatitis: 3954 images, 327 cases	AI can effectively assist in identifying key pathological features and classifying inflammatory skin conditions	[30]
**B.** **Workflow automation**	AI-based mitosis detection algorithms	Mitotic figure detection	99 digitized melanocytic lesion slides (including 10 nevoid melanomas)	Mitosis detection algorithms may be helpful in particularly challenging cases like nevoid melanoma but offer limited benefit in routine lesion interpretation	[32]
	Attention-based multiple-instance learning, transformers, saliency maps	Hotspot identification		Reduce review time and improve safety but face challenges such as spurious attention to artifacts, sensitivity–specificity trade-offs in small-lesion detection, and overconfidence on out-of-distribution detection	[12,33,34,35,36]
	DL-based cell detection (ResNet), QuPath	Objective quantification of Ki-67, Melan-A, SOX10, CD3, CD8, PD-L1	Multicenter dataset of MelanA immunostain and H&E WSIs from 464 melanocytic lesions (melanomas, melanoma in situ, and nevi) [38]	ResNet-based AI models using MelanA immunostain achieved melanoma classification performance comparable to H&E-based models, while combining both stains improved diagnostic accuracy and generalizability [38]	[37,38,39]
			20 full-thickness skin punch biopsies from 10 psoriasis patients, including 10 lesional and 10 peri-lesional unaffected skin [39]	Adobe Photoshop and QuPath showed good agreement for quantifying CD8+ T cells in psoriasis biopsies, with normalization to epidermal length providing more consistent results than area-based normalization [39]	
	WSI platforms, QuPath, AI-assisted annotation	Digital slide management and annotation			[42,43,44,45]
**C.** **Predictive analytics andprognostication**	Deep learning applied to digitized slides	Predicts disease-specific survival and risk stratification in melanoma			[49]
	Integrated histology + genomic + clinical AI	Improved personalized prognostication and treatment guidance			[50]
**D.** **Educational tools and training**	Hypertext Atlas of Dermatopathology; SlideTutor; ReportTutor	Skill refinement, diagnostic feedback, report quality improvement			[53,54,55]
	College of American pathologists (CAP) “AI Playground”	Exposure to diverse and complex cases			https://www.pathpresenter.com/cap-announces-immersive-ai-playground-powered-by-pathpresenter/ (accessed on 29 May 2026)

## 4. Methodological Considerations and Technical Aspects

Methodological considerations and key technical aspects of artificial intelligence in dermatopathology are summarized in Table 3, which highlights important factors influencing AI development and deployment in this field.

### 4.1. Data Acquisition and Curation

#### 4.1.1. Importance of High-Quality, Annotated Datasets

AI in dermatopathology relies on large, diverse, and expertly annotated datasets. High-quality annotations, ranging from slide-level labels to region-of-interest demarcations, are essential for training supervised models and validating algorithmic performance. However, annotation is time consuming and is prone to inter-observer variability, highlighting the need for standardized protocols and multi-institutional efforts to ensure reproducibility and generalizability [43].

#### 4.1.2. Whole-Slide Imaging (WSI) and Its Role

WSIs allow comprehensive evaluation of tissue architecture and cellular morphology while facilitating storage and data sharing across institutions [41]. They provide the raw material for computational pathology pipelines, though challenges such as larger file sizes, scanner variability, and image standardization remain critical consideration for multi-center research [56].

#### 4.1.3. Data Augmentation Techniques

Given the limited availability of annotated dermatopathology datasets, data augmentation is commonly employed to increase sample diversity. Techniques such as rotation, scaling, color normalization, or stain augmentation help simulate variability encountered in real-world slides [57]. These methods allow AI systems to generalize better across institutions and scanner platforms.

### 4.2. Algorithm Development and Training

#### 4.2.1. Convolutional Neural Networks (CNNs) and Their Architectures

CNNs represent the foundation of image-based AI in histopathology, as they can automatically learn spatial hierarchies of features (edges > textures > patterns > complex structures). CNNs can capture tissue morphology, cell-level structures, and tumor–stroma interactions from WSIs, and that is what makes them relevant to pathology [58]. Over time, CNN architectures have evolved to address challenges of depth, efficiency, and feature representation:

- Basic CNNs: Layers of convolution, pooling, and fully connected layers [59].
- ResNet: Introduced residual connections to enable training of very deep networks without vanishing gradients [60].
- DenseNet: Densely connected layers to improve feature reuse and efficiency [61].
- Inception/GoogLeNet: Multi-scale convolutional filters for extracting features at different resolutions [62].
- Vision transformers (ViTs): More recently used for pathology and leveraging attention mechanisms [63].

#### 4.2.2. Transfer Learning

Deep learning has played a central role in medical image classification, where multiple images are analyzed to generate a single diagnostic outcome. Since medical datasets are often smaller than those in computer vision, transfer learning has become a widely adopted strategy [58]. Transfer learning uses models pre-trained on large datasets and fine tunes them on smaller, domain-specific datasets [58]. It has become essential in digital pathology due to the scarcity and high cost of annotated WSI. By reducing computational demands and training time, transfer learning enables better generalization on limited datasets [64].

#### 4.2.3. Explainable AI (XAI) for Interpretability

XAI is essential for translating deep learning models into clinically usable tools, which allow pathologists to understand algorithmic outputs [65]. Common approaches include saliency mapping techniques, which have been used to visualize regions of histopathology slides most influential to model’s decision; multiple instance learning (MIL) frameworks, which help identify patches within WSI drive classifications such as tumor subtype; and prototype-based models, which compare new cases with representative examples [65]. Despite these advances, XAI methods can misidentify artifacts or irrelevant tissue regions. To be clinically useful, explanations must align with dermatopathological hallmarks such as epidermal changes and mitotic activity [66].

#### 4.2.4. Large Language Models (LLMs)

LLMs, a class of AI systems designed to process, interpret, and generate human-like text, are increasingly being explored beyond their traditional text-based applications [67]. A growing extension of LLMs in dermatopathology is their multimodal use, particularly image-text systems capable of interpreting clinical, macroscopic, and dermoscopic images to suggest a histopathologic diagnosis. Across three studies from Semmelweis University and related cohorts, multimodal LLMs were evaluated for skin neoplastic lesion diagnosis using clinical and dermoscopic images with standardized prompting (“Can you guess the most likely diagnosis?”). In melanoma vs. nevus classification (807 images), ChatGPT-4o showed high sensitivity—especially on dermoscopy (96.5%)—but lower specificity, while Gemini 2.0 Flash showed the opposite pattern, with higher specificity; together, they achieved near-complete case capture (96.2%) [68]. In actinic keratosis vs. squamous cell carcinoma (112 patients), ChatGPT-4o again outperformed Gemini in accuracy and sensitivity, but both models had low negative predictive value, meaning malignant cases could still be missed despite “negative” outputs [69]. In BCC detection and subtyping (772 images), newer web-based models (ChatGPT-5, Gemini 2.5 Flash, Claude Sonnet 4) showed only moderate performance, with best accuracies ranging from ~50 to 75%, depending on model and imaging type, and reduced performance in certain lesion morphologies; subtype classification was also limited [70]. Overall, across all three studies, multimodal LLMs demonstrated promising but inconsistent diagnostic ability, with better performance in sensitive screening than reliable exclusion, supporting their potential as adjunct triage tools rather than standalone diagnostic systems and highlighting the need for dermatology-specific training and rigorous validation before clinical use.

### 4.3. Validation and Performance Evaluation

#### 4.3.1. Internal vs. External Validation

Internal validation uses subsets of the same dataset for training and testing, whereas external validation uses an independent dataset (e.g., from different institutions) providing a more reliable estimate of real-world performance [71].

#### 4.3.2. Metrics

The performance of models is typically evaluated with several metrics. Accuracy measures the overall proportion of samples classified correctly. Sensitivity and specificity quantify the ability of a model to correctly identify positive and negative cases, respectively. The F1-score provides the trade-off between precision and recall, which is particularly valuable when false negatives are of clinical significance. The area under the receiver operating characteristic curve (AUC-ROC) is a threshold-independent measure of performance and is preferred in imbalanced datasets. Together, these metrics ensure that AI systems are accurate and generalizable for integration into dermatopathology workflow [72]; however, discrimination metrics alone may not fully reflect clinical utility. Increasing emphasis has therefore been placed on model calibration, which examines whether predicated probabilities correspond to true clinical risk [73]. Well-calibrated models are important in dermatopathology, since diagnostic confidence may directly influence downstream clinical decisions such as additional biopsy or re-excision. Confidence and uncertainty estimation are also recognized as critical components of AI systems, allowing algorithms to detect cases that may require expert review [34,74]. Decision curve analysis may further help determine whether AI-assisted predictions provide meaningful net clinical benefit compared with conventional diagnostic strategies [75]. System-level evaluation is also important and may include measures such as reduction in turnaround time, pathologist workload, diagnostic error rates, and workflow efficiency.

#### 4.3.3. Benchmarking Against Human Experts and Real-World Evaluation

One of the key steps in AI evaluation is comparing algorithmic performance with human experts, demonstrating whether it can achieve expert-level performance, and assess its interpretability and diagnostic confidence. In addition to retrospective testing, prospective validation and silent trails are becoming important for assessing real-world deployment. In silent trials, AI systems work in parallel with routine clinical workflows without influencing patient management, enabling investigators to evaluate safety workflow integration and potential sources of errors [76].

### 4.4. Hardware and Software Infrastructure Requirements

AI implementation requires robust computational infrastructure, including high performance servers with graphic processing units (GPUs) or tensor processing units (TPUs) capable of handling gigapixel WSIs [77]. On the software side, frameworks such as TensorFlow and PyTorch support AI model development, while tools like QuPath enable image annotation and analysis [44].

**Table 3:** Methodological considerations and technical aspects in artificial intelligence (AI) for dermatopathology.

Domain	Key Components	Key Points/Role in AI Development	Main Challenges	References
Data acquisition and curation	Annotated datasets	High-quality, expert-labeled datasets are essential for supervised learning and validation	Time-consuming annotation, inter-observer variability, lack of standardization	[43]
	Whole-slide imaging (WSI)	Enables digitization of entire histology slides, supporting computational pathology and data sharing	Large file sizes, scanner variability, cross-institution standardization	[41,56]
	Data augmentation	Rotation, scaling, color/stain normalization increase dataset diversity and model robustness	Risk of unrealistic synthetic variation, inconsistent augmentation practices	[57]
Algorithm development and training	Convolutional neural networks (CNNs) architectures	Learn hierarchical histologic features (cell → tissue → architecture); include ResNet, DenseNet, Inception	Overfitting, interpretability limitations	[58,59,60,61,62]
	Transfer learning	Uses pre-trained models to improve performance on small medical datasets	Domain shift between natural and histopathology images	[58]
	Explainable AI (XAI)	Saliency maps, multiple instance learning, prototype models improve interpretability	May misidentify artifacts; limited clinical alignment with histopathologic patterns	[65,66]
	Large language models (multimodal)	Combine clinical + dermoscopic image interpretation; show promise in screening and triage	Variable performance, low specificity, not reliable standalone tools	[67,68,69,70]
Validation and performance evaluation	Internal vs. external validation	External validation better reflects real-world generalizability	Limited availability of independent datasets	[71]
	Performance metrics	Accuracy, sensitivity, specificity, F1-score, and receiver operating characteristic area under the curve assess classification performance	Metrics alone may not reflect clinical utility	[72]
	Calibration and uncertainty	Ensures predicted probabilities reflect true risk; supports clinical decision-making	Often under-reported in studies	[73,74]
	Decision curve analysis	Evaluates net clinical benefit vs. traditional workflows	Requires robust clinical modeling assumptions	[75]
	Human benchmarking	Comparison with dermatopathologists establishes expert-level performance	Variability among human experts	
	Prospective and silent trials	Evaluate real-world integration without influencing care	Logistically complex, resource-intensive	[76]
Infrastructure requirements	Hardware	Graphic processing unit (GPU) or tensor processing unit (TPU) systems required for WSI processing and deep learning	High cost, scalability limitations	[77]
	Software tools	TensorFlow, PyTorch, QuPath (https://qupath.github.io) for model development and annotation	Interoperability and workflow integration challenges	[44]

## 5. Challenges and Limitations

### 5.1. Data-Related Challenges

One of the data-related challenges in AI is that large, diverse, and well-annotated histopathology datasets are still not widely available in dermatopathology [71]. In addition, rare entities such as adnexal tumors are underrepresented, which limits generalizability [31]. As a result, models may demonstrate strong overall accuracy driven by common entities but perform substantially worse when encountering underrepresented subtypes.

Unequal representation of certain demographic groups, skin tones, and skin lesions is another challenge in applying AI to dermatopathology [71]. Lesions in darker Fitzpatric skin types may show different pigmentation patterns and tumor visibility compared with lighter skin, which can affect extraction and downstream classification. For instance, subtle melanocytic proliferation may be more difficult to detect in heavily pigmented backgrounds, potentially reducing sensitivity in these populations when models are not adequately trained on diverse skin tones. Technical variability further compounds these issues. Variations in staining intensity, tissue processing, and scanner resolution across different laboratories will affect color and texture features, leading to reduced model reliability and diagnostic errors [78].

Future studies should report model performance stratified by clinically relevant subgroups, including patient demographic factors, lesion subtype (e.g., common versus rare tumors), anatomical site, institution, and scanner type. Such stratified reporting would help identify hidden performance disparities and better define the boundaries of model generalizability in dermatopathology AI applications.

### 5.2. Algorithmic and Technical Challenges

Application of AI faces many technical challenges. The “Black Box” problem refers to the limited interpretability of deep learning models; pathologists sometimes cannot fully understand why an algorithm reaches a certain decision, which hinders clinical trust and accuracy [66]. Efforts such as XAI are essential for translating deep learning models into clinically usable tools [65].

Another major challenge is generalizability, in which models trained on data from one institution often perform poorly on external datasets. This highlights the need for multi-institutional and diverse datasets. Lastly, AI systems require computational resources, which may limit adoption in smaller institutions.

### 5.3. Integration and Adoption Challenges

Regulatory approval and standardization remain major obstacles, as there are no universally accepted guidelines for validating and approving medical AI tools. Pathologist trust and acceptance are also critical; many professionals express concerns about overreliance on AI and the potential job loss. Additionally, interoperability with existing workflows, such as integration with laboratory information systems (LISs), is essential for practical use but technically complex [79]. Finally, the cost of implementation and maintenance can limit the adoption of AI in smaller institutions, slowing widespread AI adoption.

## 6. Ethical and Legal Considerations

When diagnostic tools powered by AI are used and a diagnostic error occurs, it becomes challenging to allocate responsibility [80]. While clinicians are held responsible for diagnostic decisions, liability may also extend to AI developers or device manufacturers if system flaws are identified, underscoring the need for clear regulations to guide the safe and ethical application of AI in diagnostic medicine.

Ethical oversight is essential through the development of AI tools in dermatopathology. Lalmalani et al. [71] emphasized several ethical considerations, including the need for developers to obtain appropriate research and ethics approvals and to ensure the responsible use of patient data, particularly when such data are used for research purposes.

Algorithmic bias can lead to reduced diagnostic accuracy in minority populations. Ensuring fairness requires inclusion of diverse datasets and transparent validation across populations.

Integration of AI in dermatopathology is reshaping professional roles and requires new skills. Rather than replacing humans, AI serves as a tool that helps in enhancing diagnostic accuracy and workflow management. Training programs must adapt to include education on AI and on how to use and evaluate its output [13].

## 7. Future Directions and Emerging Trends

Although several emerging technologies may influence dermatopathology, two areas are likely to have the greatest near-term clinical impact: multimodal and foundation AI models and federated learning integrated into digital pathology workflows. These priorities address the current limitations of isolated image-based algorithms, including limited generalizability, lack of clinical context, and restricted access to diverse training datasets.

### 7.1. Multi-Modal AI and Foundation Models

Multimodal AI frameworks show promise for complex diseases such as cutaneous lymphomas and melanomas, where clinical, imaging, proteomic, and genomic information add crucial context. Combining histology with molecular data can improve prediction of tumor behavior and treatment response. Foundation models and vision–language models (VLMs) are expected to play a central role in this transition. Vision–language models (VLMs) can process both images and text simultaneously, linking visual features to descriptive annotations. In dermatopathology, this can enable automatic slide annotation, region-of-interest highlighting, and natural language explanations of histopathologic findings [81]. In parallel, large language model (LLM)-based assistants may support dermatopathologists by generating draft reports, suggesting ancillary studies, answering diagnostic questions, and reducing cognitive workload.

### 7.2. Federated Learning and Workflow Integration

Another major priority is federated learning and collaborative multi-institutional datasets, which address current limitations related to small datasets, poor generalizability, and patient privacy. Federated learning allows AI models to be trained across institutions without transferring patient data [72]. These advances will support broader integration of AI into digital pathology workflows, including slide scanning, quality control, image analysis, case triage, and teledermatopathology, ultimately improving efficiency and access to subspecialty expertise [73].

Additional emerging areas include chatbots and diagnostic assistants, explainable AI, adaptive continuous-learning systems, and AI-driven personalized medicine approaches.

## 8. Conclusions

AI is increasingly influencing dermatopathology by enhancing diagnostic accuracy, improving workflow efficiency, and supporting education and research. Current applications include assistance in diagnosing melanocytic lesions, non-melanoma skin cancer, and inflammatory skin disease, as well as automation of routine tasks and prognostic prediction. Despite its promise, AI integration faces significant challenges, including algorithm development, model validation, and generalizability. Addressing these issues requires close collaboration among dermatopathologists and data scientists to ensure that AI tools are safe and reliable. Looking ahead, AI holds the potential to be fully integrated into routine dermatopathology practice, advancing the standard of care and improving patient outcomes through a collaborative model that combines algorithmic precision with human expertise.

## Figures and Tables

**Table 1:** Advantages and challenges of artificial intelligence (AI) integration in clinical dermatopathology practice.

Aspect	Advantages	Challenges
Diagnostic accuracy	AI algorithms can identify subtle histopathologic patterns, improve lesion classification, and reduce diagnostic errors.	Performance may decline when applied to external datasets due to limited generalizability and dataset bias.
Efficiency and workflow	AI can rapidly analyze large numbers of slides, assist in triaging cases, and reduce turnaround times in high-volume practices.	Integration into existing laboratory workflows and digital pathology systems can be technically complex and costly.
Standardization	AI-assisted interpretation may improve consistency across institutions and reduce inter-observer variability.	Lack of standardized validation protocols and regulatory frameworks may limit widespread implementation.
Workforce support	AI may help alleviate dermatopathologist shortages by functioning as a second reader and automating repetitive tasks.	Overreliance on AI could reduce independent diagnostic skills and may raise concerns regarding replacement of human expertise.
Educational applications	AI tools can support students and resident education, image annotation, and training through pattern recognition assistance.	Training datasets may contain labeling errors or lack diversity. AI could lead to reduced independent diagnostic reasoning.
Accessibility	AI-assisted systems may improve access to expert-level diagnostic support in underserved or remote regions.	Implementation requires substantial digital infrastructure, including slide scanners and storage capacity.

## Data Availability

No new data were created or analyzed in this study. Data sharing is not applicable to this article.

## References

[1] Hay R.J., Johns N.E., Williams H.C., Bolliger I.W., Dellavalle R.P., Margolis D.J., Marks R., Naldi L., Weinstock M.A., Wulf S.K. (2014). The global burden of skin disease in 2010: An analysis of the prevalence and impact of skin conditions. J. Investig. Dermatol..

[2] Cramer S.F. (1997). Interobserver variability in dermatopathology. Arch. Dermatol..

[3] Roky A.H., Islam M.M., Ahasan A.M.F., Mostaq M.S., Mahmud M.Z., Amin M.N., Mahmud M.A. (2025). Overview of skin cancer types and prevalence rates across continents. Cancer Pathog. Ther..

[4] Walsh E., Orsi N.M. (2024). The current troubled state of the global pathology workforce: A concise review. Diagn. Pathol..

[5] Dave M., Patel N. (2023). Artificial intelligence in healthcare and education. Br. Dent. J..

[6] Eapen B.R. (2020). Artificial Intelligence in Dermatology: A Practical Introduction to a Paradigm Shift. Indian Dermatol. Online J..

[7] Sadr H., Nazari M., Khodaverdian Z., Farzan R., Yousefzadeh-Chabok S., Ashoobi M.T., Hemmati H., Hendi A., Ashraf A., Pedram M.M. (2025). Unveiling the potential of artificial intelligence in revolutionizing disease diagnosis and prediction: A comprehensive review of machine learning and deep learning approaches. Eur. J. Med. Res..

[8] Wells A., Patel S., Lee J.B., Motaparthi K. (2021). Artificial intelligence in dermatopathology: Diagnosis, education, and research. J. Cutan. Pathol..

[9] Ramesh A.N., Kambhampati C., Monson J.R., Drew P.J. (2004). Artificial intelligence in medicine. Ann. R. Coll. Surg. Engl..

[10] Kaul V., Enslin S., Gross S.A. (2020). History of artificial intelligence in medicine. Gastrointest. Endosc..

[11] Potter B., Ronan S.G. (1987). Computerized dermatopathologic diagnosis. J. Am. Acad. Dermatol..

[12] Cazzato G., Rongioletti F. (2024). Artificial intelligence in dermatopathology: Updates, strengths, and challenges. Clin. Dermatol..

[13] Polesie S., McKee P.H., Gardner J.M., Gillstedt M., Siarov J., Neittaanmäki N., Paoli J. (2020). Attitudes Toward Artificial Intelligence Within Dermatopathology: An International Online Survey. Front. Med..

[14] Hekler A., Utikal J.S., Enk A.H., Berking C., Klode J., Schadendorf D., Jansen P., Franklin C., Holland-Letz T., Krahl D. (2019). Pathologist-level classification of histopathological melanoma images with deep neural networks. Eur. J. Cancer.

[15] Brinker T.J., Schmitt M., Krieghoff-Henning E.I., Barnhill R., Beltraminelli H., Braun S.A., Carr R., Fernandez-Figueras M.T., Ferrara G., Fraitag S. (2022). Diagnostic performance of artificial intelligence for histologic melanoma recognition compared to 18 international expert pathologists. J. Am. Acad. Dermatol..

[16] Cazzato G., Massaro A., Colagrande A., Trilli I., Ingravallo G., Casatta N., Lupo C., Ronchi A., Franco R., Maiorano E. (2023). Artificial Intelligence Applied to a First Screening of Naevoid Melanoma: A New Use of Fast Random Forest Algorithm in Dermatopathology. Curr. Oncol..

[17] Hekler A., Utikal J.S., Enk A.H., Solass W., Schmitt M., Klode J., Schadendorf D., Sondermann W., Franklin C., Bestvater F. (2019). Deep learning outperformed 11 pathologists in the classification of histopathological melanoma images. Eur. J. Cancer.

[18] Elshahawy M., Elnemr A., Oproescu M., Schiopu A.-G., Elgarayhi A., Elmogy M.M., Sallah M. (2023). Early Melanoma Detection Based on a Hybrid YOLOv5 and ResNet Technique. Diagnostics.

[19] Duschner N., Baguer D.O., Schmidt M., Griewank K.G., Hadaschik E., Hetzer S., Wiepjes B., Le’Clerc Arrastia J., Jansen P., Maass P. (2023). Applying an artificial intelligence deep learning approach to routine dermatopathological diagnosis of basal cell carcinoma. J. Dtsch. Dermatol. Ges..

[20] Yin W., Zhou D., Nie R. (2023). DI-UNet: Dual-branch interactive U-Net for skin cancer image segmentation. J. Cancer Res. Clin. Oncol..

[21] Nogales A., Garrido M.C., Guitian A., Rodriguez-Peralto J.-L., Villanueva C.P., Díaz-Prieto D., García-Tejedor Á.J. (2025). A Hybrid Artificial Intelligence Framework for Melanoma Diagnosis Using Histopathological Images. Technologies.

[22] Cruz-Roa A.A., Arevalo Ovalle J.E., Madabhushi A., González Osorio F.A. A deep learning architecture for image representation, visual interpretability and automated basal-cell carcinoma cancer detection. Proceedings of the International Conference on Medical Image Computing and Computer-Assisted Intervention MICCAI 2013.

[23] Lan X., Guo G., Wang X., Yan Q., Xue R., Li Y., Zhu J., Dong Z., Wang F., Li G. (2024). Differentiation and risk stratification of basal cell carcinoma with deep learning on histopathologic images and measuring nuclei and tumor microenvironment features. Skin. Res. Technol..

[24] Levy J.J., Davis M.J., Chacko R.S., Davis M.J., Fu L.J., Goel T., Pamal A., Nafi I., Angirekula A., Suvarna A. (2024). Intraoperative margin assessment for basal cell carcinoma with deep learning and histologic tumor mapping to surgical site. npj Precis. Oncol..

[25] Tighe D., Tekeli K., Gouk T., Smith J., Ho M., Moody A., Walsh S., Provost S., Freitas A. (2023). Machine learning methods applied to audit of surgical margins after curative surgery for facial (non-melanoma) skin cancer. Br. J. Oral Maxillofac. Surg..

[26] Bittner G.C., Cerci F.B., Kubo E.M., Tolkachjov S.N. (2021). Mohs micrographic surgery: A review of indications, technique, outcomes, and considerations. An. Bras. Dermatol..

[27] Davis M.J., Srinivasan G., Chacko R., Chen S., Suvarna A., Vaickus L.J., Torres V.C., Hodge S., Chen E.Y., Preum S. (2024). A deep learning algorithm to detect cutaneous squamous cell carcinoma on frozen sections in Mohs micrographic surgery: A retrospective assessment. Exp. Dermatol..

[28] Della Mura M., Sorino J., Colagrande A., Daruish M., Ingravallo G., Massaro A., Cazzato G., Lupo C., Casatta N., Ribatti D. (2025). Artificial Intelligence in the Histopathological Assessment of Non-Neoplastic Skin Disorders: A Narrative Review with Future Perspectives. Med. Sci..

[29] Pal A., Garain U., Chandra A., Chatterjee R., Senapati S. (2018). Psoriasis skin biopsy image segmentation using Deep Convolutional Neural Network. Comput. Methods Programs Biomed..

[30] Bao Y., Zhang J., Zhang Q., Chang J., Lu D., Fu Y. (2021). Artificial Intelligence-Aided Recognition of Pathological Characteristics and Subtype Classification of Superficial Perivascular Dermatitis. Front. Med..

[31] Jansen P., Arrastia J.L., Baguer D.O., Schmidt M., Landsberg J., Wenzel J., Emberger M., Schadendorf D., Hadaschik E., Maass P. (2024). Deep learning based histological classification of adnex tumors. Eur. J. Cancer.

[32] Sturm B., Creytens D., Smits J., Ooms A.H.A.G., Eijken E., Kurpershoek E., Küsters-Vandevelde H.V.N., Wauters C., Blokx W.A.M., van der Laak J.A.W.M. (2022). Computer-Aided Assessment of Melanocytic Lesions by Means of a Mitosis Algorithm. Diagnostics.

[33] Su Z., Rezapour M., Sajjad U., Niu S., Gurcan M.N., Niazi M.K.K. (2024). Cross-Attention-Based Saliency Inference for Predicting Cancer Metastasis on Whole Slide Images. IEEE J. Biomed. Health Inform..

[34] Dolezal J.M., Srisuwananukorn A., Karpeyev D., Ramesh S., Kochanny S., Cody B., Mansfield A.S., Rakshit S., Bansal R., Bois M.C. (2022). Uncertainty-informed deep learning models enable high-confidence predictions for digital histopathology. Nat. Commun..

[35] Cai L., Huang S., Zhang Y., Lu J., Zhang Y. (2025). AttriMIL: Revisiting attention-based multiple instance learning for whole-slide pathological image classification from a perspective of instance attributes. Med. Image Anal..

[36] Linmans J., Elfwing S., van der Laak J., Litjens G. (2023). Predictive uncertainty estimation for out-of-distribution detection in digital pathology. Med. Image Anal..

[37] Brogård M.B., Steiniche T., Lade-Keller J., Wandler A., Christensen K.B., Georgsen J.B., Nielsen P.S. (2025). Digital quantification of Ki67 and PRAME in challenging melanocytic lesions—A novel diagnostic tool. Pathol. Res. Pract..

[38] Wies C., Schneider L., Haggenmüller S., Bucher T.C., Hobelsberger S., Heppt M.V., Ferrara G., Krieghoff-Henning E.I., Brinker T.J. (2024). Evaluating deep learning-based melanoma classification using immunohistochemistry and routine histology: A three center study. PLoS ONE.

[39] Emmanuel T., Brent M.B., Iversen L., Johansen C. (2022). Quantification of Immunohistochemically Stained Cells in Skin Biopsies. Dermatopathology.

[40] Baxi V., Lee G., Duan C., Pandya D., Cohen D.N., Edwards R., Chang H., Li J., Elliott H., Pokkalla H. (2022). Association of artificial intelligence-powered and manual quantification of programmed death-ligand 1 (PD-L1) expression with outcomes in patients treated with nivolumab ± ipilimumab. Mod. Pathol..

[41] Al-Janabi S., Huisman A., Vink A., Leguit J., Offerhaus G.J.A., ten Kate J.F.W., van Dijk R., van Diest P.J. (2012). Whole slide images for primary diagnostics in dermatopathology: A feasibility study. J. Clin. Pathol..

[42] Weinstein R.S., Graham A.R., Richter L.C., Barker G.P., Krupinski E.A., Lopez A.M., Erps K.A., Bhattacharyya A.K., Yagi Y., Gilbertson J.R. (2009). Overview of telepathology, virtual microscopy, and whole slide imaging: Prospects for the future. Hum. Pathol..

[43] Tschandl P., Rinner C., Apalla Z., Argenziano G., Codella N., Halpern A., Janda M., Lallas A., Longo C., Malvehy J. (2020). Human-computer collaboration for skin cancer recognition. Nat. Med..

[44] Bankhead P., Loughrey M.B., Fernández J.A., Dombrowski Y., McArt D.G., Dunne P.D., McQuaid S., Gray R.T., Murray L.J., Coleman H.G. (2017). QuPath: Open source software for digital pathology image analysis. Sci. Rep..

[45] Amgad M., Elfandy H., Hussein H., Atteya L.A., Elsebaie M.A.T., Abo Elnasr L.S., Sakr R.A., Salem H.S.E., Ismail A.F., Saad A.M. (2019). Structured crowdsourcing enables convolutional segmentation of histology images. Bioinformatics.

[46] Liu Y., He Q., Duan H., Shi H., Han A., He Y. (2022). Using Sparse Patch Annotation for Tumor Segmentation in Histopathological Images. Sensors.

[47] Tavolara T.E., Su Z., Gurcan M.N., Niazi M.K.K. (2023). One label is all you need: Interpretable AI-enhanced histopathology for oncology. Semin. Cancer Biol..

[48] Comes M.C., Fucci L., Mele F., Bove S., Cristofaro C., De Risi I., Fanizzi A., Milella M., Strippoli S., Zito A. (2022). A deep learning model based on whole slide images to predict disease-free survival in cutaneous melanoma patients. Sci. Rep..

[49] Li X., Yu X., Tian D., Liu Y., Li D. (2023). Exploring and validating the prognostic value of pathomics signatures and genomics in patients with cutaneous melanoma based on bioinformatics and deep learning. Med. Phys..

[50] Peng Y., Chu Y., Chen Z., Zhou W., Wan S., Xiao Y., Zhang Y., Li J. (2020). Combining texture features of whole slide images improves prognostic prediction of recurrence-free survival for cutaneous melanoma patients. World J. Surg. Oncol..

[51] Zehra T., Parwani A., Abdul-Ghafar J., Ahmad Z. (2023). A suggested way forward for adoption of AI-Enabled digital pathology in low resource organizations in the developing world. Diagn. Pathol..

[52] Shahriari N., Grant-Kels J., Murphy M.J. (2017). Dermatopathology education in the era of modern technology. J. Cutan. Pathol..

[53] Feit J., Kempf W., Jedlicková H., Burg G. (2005). Hypertext atlas of dermatopathology with expert system for epithelial tumors of the skin. J. Cutan. Pathol..

[54] Crowley R.S., Medvedeva O. (2006). An intelligent tutoring system for visual classification problem solving. Artif. Intell. Med..

[55] Crowley R.S., Tseytlin E., Jukic D. (2005). ReportTutor—An intelligent tutoring system that uses a natural language interface. AMIA Annu. Symp. Proc..

[56] Mukhopadhyay S., Feldman M.D., Abels E., Ashfaq R., Beltaifa S., Cacciabeve N.G., Cathro H.P., Cheng L., Cooper K., Dickey G.E. (2018). Whole Slide Imaging Versus Microscopy for Primary Diagnosis in Surgical Pathology: A Multicenter Blinded Randomized Noninferiority Study of 1992 Cases (Pivotal Study). Am. J. Surg. Pathol..

[57] Tellez D., Litjens G., Bándi P., Bulten W., Bokhorst J.M., Ciompi F., van der Laak J. (2019). Quantifying the effects of data augmentation and stain color normalization in convolutional neural networks for computational pathology. Med. Image Anal..

[58] Khosravi P., Kazemi E., Imielinski M., Elemento O., Hajirasouliha I. (2018). Deep Convolutional Neural Networks Enable Discrimination of Heterogeneous Digital Pathology Images. eBioMedicine.

[59] Colliot O. (2023). Machine Learning for Brain Disorders.

[60] Xu W., Fu Y.L., Zhu D. (2023). ResNet and its application to medical image processing: Research progress and challenges. Comput. Methods Programs Biomed..

[61] Zhou T., Ye X., Lu H., Zheng X., Qiu S., Liu Y. (2022). Dense Convolutional Network and Its Application in Medical Image Analysis. Biomed. Res. Int..

[62] Coy H., Hsieh K., Wu W., Nagarajan M.B., Young J.R., Douek M.L., Brown M.S., Scalzo F., Raman S.S. (2019). Deep learning and radiomics: The utility of Google TensorFlow™ Inception in classifying clear cell renal cell carcinoma and oncocytoma on multiphasic CT. Abdom. Radiol..

[63] Park S.Y., Ayana G., Wako B.D., Jeong K.C., Yoon S.-D., Choe S.-w. (2025). Vision Transformers for Low-Quality Histopathological Images: A Case Study on Squamous Cell Carcinoma Margin Classification. Diagnostics.

[64] Bungărdean R.M., Şerbănescu M.S., Streba C.T., Crişan M. (2021). Deep learning with transfer learning in pathology. Case study: Classification of basal cell carcinoma. Rom. J. Morphol. Embryol..

[65] van der Velden B.H.M., Kuijf H.J., Gilhuijs K.G.A., Viergever M.A. (2022). Explainable artificial intelligence (XAI) in deep learning-based medical image analysis. Med. Image Anal..

[66] Plass M., Kargl M., Kiehl T.R., Regitnig P., Geißler C., Evans T., Zerbe N., Carvalho R., Holzinger A., Müller H. (2023). Explainability and causability in digital pathology. J. Pathol. Clin. Res..

[67] Shah A., Wahood S., Guermazi D., Brem C.E., Saliba E. (2024). Skin and Syntax: Large Language Models in Dermatopathology. Dermatopathology.

[68] Boostani M., Lallas A., Goldust M., Nádudvari N., Lőrincz K., Bánvölgyi A., Holló P., Wikonkál N.M., Paragh G., Kiss N. (2025). Diagnostic performance of multimodal large language models in distinguishing melanoma from nevi in clinical and dermoscopic images. JAAD Int..

[69] Boostani M., Pellacani G., Goldust M., Nádudvari N., Rátky D., Cantisani C., Lőrincz K., Bánvölgyi A., Wikonkál N.M., Paragh G. (2026). Diagnosing Actinic Keratosis and Squamous Cell Carcinoma With Large Language Models From Clinical Images. Int. J. Dermatol..

[70] Boostani M., Zouboulis C.C., Pellacani G., Navarrete-Dechent C., Boussingault L., Kiss T., Goldfarb N., Cantisani C., Nádudvari N., Bánvölgyi A. (2026). ChatGPT, Gemini, and Claude in clinical and dermoscopic image analysis of basal cell carcinoma and its common mimickers: A comparative performance analysis. JID Innov..

[71] Lalmalani R.M., Lim C.X.Y., Oh C.C. (2025). Artificial intelligence in dermatopathology: A systematic review. Clin. Exp. Dermatol..

[72] Flores J., Misra R., Shah B., Williams Y., Haghighat B., Miranda G., Jani P., Frasier K. (2025). Artificial Intelligence and Machine Learning Transforming Dermatopathology with Diagnosis and Predictive Analytics. Dermis.

[73] Van Calster B., McLernon D.J., van Smeden M., Wynants L., Steyerberg E.W. (2019). Calibration: The Achilles heel of predictive analytics. BMC Med..

[74] Olsson H., Kartasalo K., Mulliqi N., Capuccini M., Ruusuvuori P., Samaratunga H., Delahunt B., Lindskog C., Janssen E.A.M., Blilie A. (2022). Estimating diagnostic uncertainty in artificial intelligence assisted pathology using conformal prediction. Nat. Commun..

[75] Vickers A.J., Elkin E.B. (2006). Decision curve analysis: A novel method for evaluating prediction models. Med. Decis. Mak..

[76] Kwong J.C.C., Erdman L., Khondker A., Skreta M., Goldenberg A., McCradden M.D., Lorenzo A.J., Rickard M. (2022). The silent trial—The bridge between bench-to-bedside clinical AI applications. Front. Digit. Health.

[77] Landvater R.E., Balis U. (2025). Iris: A Next Generation Digital Pathology Rendering Engine. J. Pathol. Inform..

[78] Jartarkar S.R. (2023). Artificial intelligence: Its role in dermatopathology. Indian J. Dermatol. Venereol. Leprol..

[79] Tizhoosh H.R., Pantanowitz L. (2018). Artificial Intelligence and Digital Pathology: Challenges and Opportunities. J. Pathol. Inform..

[80] Cestonaro C., Delicati A., Marcante B., Caenazzo L., Tozzo P. (2023). Defining medical liability when artificial intelligence is applied on diagnostic algorithms: A systematic review. Front. Med..

[81] Gilal N.U., Zegour R., Al-Thelaya K., Özer E., Agus M., Schneider J., Boughorbel S. (2025). PathVLM-Eval: Evaluation of open vision language models in histopathology. J. Pathol. Inform..

